# Morphometric and Allozyme Variation in *Culex tritaeniorhynchus* Mosquito Populations from India

**DOI:** 10.1673/031.010.13801

**Published:** 2010-08-23

**Authors:** Phul Chand Kanojia, Mandar S. Paingankar, Avinash A. Patil, Mangesh D. Gokhale, Dileep N. Deobagkar

**Affiliations:** ^1^ Department of Medical Entomology and Zoology, National Institute of Virology, 20-A, Dr. Ambedkar Road, Pune 411001, India; ^2^ Molecular Biology Research Laboratory, Department of Zoology, University of Pune, Pune 411007, India

**Keywords:** discriminant factor analysis, genetic variation, Japanese Encephalitis vector, population variation, principle component analysis

## Abstract

Four populations of *Culex tritaeniorhynchus* (Giles) (Diptera: Culicidae), collected from Bellary, Cuddalore, Pune, and the Microbial Containment Complex laboratory culture in India were analyzed for morphological and allozyme variation. Multivariate analysis based on eight morphological characteristics and three morphometric indices was used to investigate the morphological variations among the four populations. Principal component analysis of the data suggested that siphon, saddle, and anal gills related variables were most important. Discriminant factor analysis of morphological data revealed that the four populations form significantly different clusters which can be differentiated from each other based on siphon, saddle, and pectin teeth related variables. Allozyme electrophoresis of the four populations revealed that the mean heterozygosity per locus value had high variation, ranging from 0.0879 to 1.794. F_st_ values between 0 and 0.519 suggested genetic differentiation within these populations. F_is_ values ranged from 0 to 1 with most of the values closer to 1. The allelic frequencies and Nei's genetic identity values showed that genetic differences between populations were small, but significant. Some of the morphological and allozyme variations in the *Cx. tritaeniorhynchus* populations could be partly attributed to the environmental conditions. The findings suggested that transition of morphological characters and allozyme variations in *Cx. tritaeniorhynchus* populations seem to be consequences of influence and selection by the environmental conditions. These results indicated that populations of *Cx. tritaeniorhynchus* in non-endemic areas of Japanese encephalitis (JE) virus infection have higher adaptability as compared to endemic areas of JE infection.

## Introduction

*Culex tritaeniorhynchus* (Giles) (Diptera: Culicidae) has emerged as an important vector of Japanese encephalitis (JE) virus in east, southeast, and south Asia (Reuben et al. 1994). While *Cx. tritaeniorhynchus* is present throughout India, JE is endemic only in seven states (Andhra Pradesh, Assam, Bihar, Karnataka, Tamilnadu, Uttar Pradesh, and West Bengal). The change in the pattern of JE virus transmission in some parts of India from epidemic to endemic is partly correlated with the establishment of *Cx. tritaeniorhynchus* in these areas. The ability of *Cx. tritaeniorhynchus* to transmit and spread JE across India has become a topic of concern. Understanding the role of *Cx. tritaeniorhynchus* as a zoonotic and epizootic vector of JE is of primary importance in the understanding the JE epidemiology. Differences in transmission and vector competence of *Cx. tritaeniorhynchus* for JE provide reasons for investigation of variations in the populations of this species. However, in spite of its epidemiological importance in JE transmission, few studies have been done on *Cx. tritaeniorhynchus.* Neither the evolutionary history nor the population dynamics of this species is well understood. Investigation of different levels of genetic and morphological variation among the populations of *Cx. tritaeniorhynchus* will be important in designing vector control strategies. Considering repeated cases of JE infection, comparison of population variations in *Cx. tritaeniorhynchus* of endemic versus non-endemic areas of JE infection will be important in designing improved vector control measures. The present study attempted to address some of the above concerns by studying morphological and genetic differences between four populations of *Cx. tritaeniorhynchus* belonging to JE endemic and non-endemic areas.

**Figure 1.**
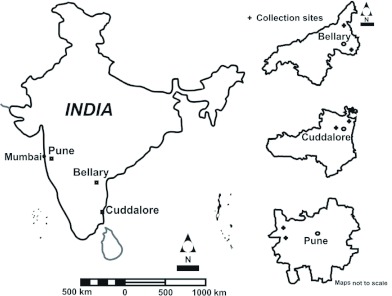
The sampling map. 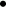 indicates sampling location. High quality figures are available online.

The description of morphological and genetic structures of wild populations of *Cx. tritaeniorhynchus* provides insights into how past and contemporary species were influenced by evolutionary forces (Hutchinson and Templeton 1999; Epperson 2005). Anatomical and morphometric measurements are useful to separate cryptic species and allow better understanding of the morphophysiological and ecological adaptation of organisms to the environment. In the case of mosquitoes, chetotaxy of fourth-instar larva and morphological characters of the male genetalia are the recommended characters for species identification (Faran 1980). However, these characters have also been used in understanding population variation (Manguin et al 1999). Very few studies have targeted population differences of *Cx. tritaeniorhynchus* at the morphological level. To the authors' knowledge there is only one study on population differences in the morphology of this species (Fakoorziba and Vijayan 2003).

The distribution and extent of species and genetic variation are related to factors such as taxonomic status (Gitzendanner and Soltis 2000), life history (Loveless and Hamrick 1984), and biogeographic history. Several studies on genetic variation in mosquito populations were carried out in *Culicidae* (Cornel et al 2003; Cui et al 2007; Manguin et al. 1999). However, studies of genetic variation in *Cx. tritaeniorhynchus* populations are scarce. Here, multilocus enzyme electrophoresis was used as a preliminary tool for assessing genetic variability and differentiation within the *Cx. tritaeniorhynchus.*

Bellary, Cuddalore, Pune, and the Microbial Containment Complex (MCC) laboratory culture in Pune were chosen as study sites because Bellary, Cuddalore and Gorakhpur are endemic and because Pune is non-endemic to JE infection. In the MCC laboratory culture, *Cx. tritaeniorhynchus* were collected from Gorakhpur and maintained for the last 12 years without supplementation from field collections. The four populations also show some variation in behavior. *Cx. tritaeniorhynchus* is well known for its exophilic nature (Reuben 1968); however, the Bellary population shows a high degree of endophilism (Kanojia and Geevargese 2004).

In this study, the question of whether the four populations differ from each other in morphological and/or genetic structure was addressed. It was hypothesized that there is no significant difference between the morphology and isoenzyme profiles of JE endemic populations (Bellary and Cuddalore) and JE non-endemic (Pune) populations. Discriminant factor analysis (DFA) and principle component analysis (PCA) were performed in order to select an aggregate of morphological characters which collectively differentiated the four populations of *Cx. tritaeniorhynchus.* Multilocus enzyme electrophoresis was used as a preliminary tool to evaluate isoenzymic, genetic, and population variability in *Cx. tritaeniorhynchus.* The importance of the study to evaluate levels of genetic diversity of *Cx. tritaeniorhynchus* populations across India was also discussed.

## Materials and Methods

Adult and larvae *Cx. tritaeniorhynchus* were sampled from four populations (Table 1) to examine the possibility of population differences in endemic and non-endemic areas of JE virus infection. Bellary and Cuddalore are endemic areas of JE virus infection, and Pune is non-endemic to JE virus infections.

**Table 1.**
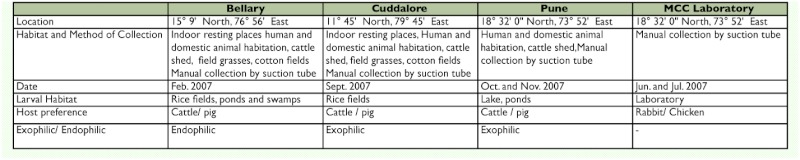
Location, Date, method of capture and behavior of *Culex tritaeniorhynchus* populations

**Table 2.**
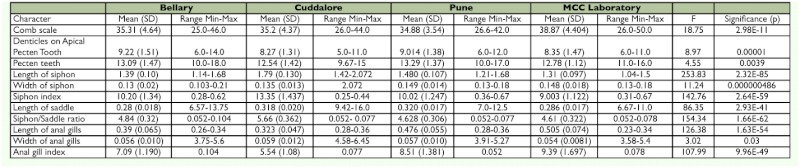
Results of one-way ANOVA for comparing means of morphological characters in four populations of *Cx. tritaeniorhynchus*

Larvae were collected from temporary and semi-permanent groundwater pools, fields, and water storage containers in houses. The MCC laboratory population was maintained in the Microbial Containment Complex (MCC) at the National Institute of Virology (NIV) in Pune for the last 12 years. The population was self-mating and fed on blood meal. The colony was not supplemented with field collections and was kept in a separate room to avoid contamination between laboratory colonies. Colonies of each population were also maintained at NIV Pune. Individuals of *Cx. tritaeniorhynchus* were identified using the keys of Reuben et al. (1994). Voucher specimens were deposited in the museum of the National Institute of Virology.

### Morphological analysis

Eight morphological characters and three ratios were scored for morphological analysis of fourth-instar larvae (Table 2). Siphon index, anal gill index, and siphon saddle ratio are used in traditional taxonomy to distinguish between species and subspecies. However, Fakoorziba and Vijayan (2003) have used these ratios to distinguish between different populations. The characters of fourth-instar larvae were measured using micrometric oculars; the least count was 0.01mm.

### Electrophoresis and detection of enzyme activity

The protein extraction for a single mosquito was prepared using a battery-operated homogenizer in a 50 µl homogenization buffer (20% Sucrose, 0.1*M* Tris-Borate, trace of bromophenol blue as a tracking dye at pH 6.8) on ice for one minute. Supernatants were collected by centrifugation at 10000g, 4° C for 5 min. Electrophoresis was carried out on a 7 cm long, 1 mm thick, 12.5% non-denaturating Polyacrylamide gel at 5° C with a constant current of 20mA (Biorad electrophoresis system) until the tracking dye (bromophenol blue) reached the bottom of the gel (approx. 90–120 min). Staining was carried out for seven different allozymes (Black and Munstermann 1996; Pasteur et al 1988) (Table 3).

**Table 3.**
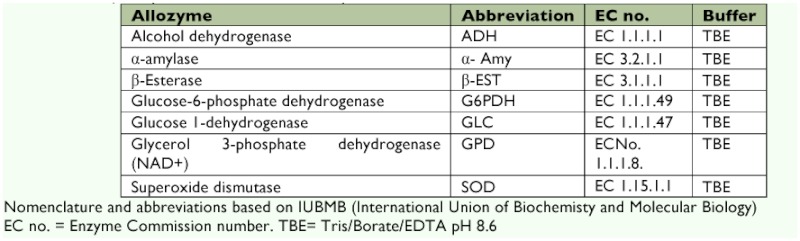
Allozyme systems resolved in this study.

### Analysis of data

One factor ANOVA was performed to evaluate the morphological differences in the four populations of *Cx. tritaeniorhynchus.* Pair-wise comparison of each characteristic was carried out using a *t-*test with the Bonferroni adjustment to the probabilities (as 66 comparisons were made, 0.05/66 = 0.000758 was used as the cutoff value). The data was analyzed using multivariate statistics. Multivariate analysis offered the advantage of taking into account all the variables in a single analysis, thus making it possible to assess variation in the morphological characters of larvae from different collection sites. Principal component analysis (PCA) was done to create uncorrelated principal components from the original variables. Factor scores estimated the actual values of individual observations for the factors, and correlation between variables and factors was called factor loading. The first two principal factors, which explained maximum variation in the data, were analyzed to understand the variation in the morphology of different individuals. Discriminant factor analysis (DFA) was done to find the variables which were most useful for discriminating between individuals of different populations.

**Table 4.**
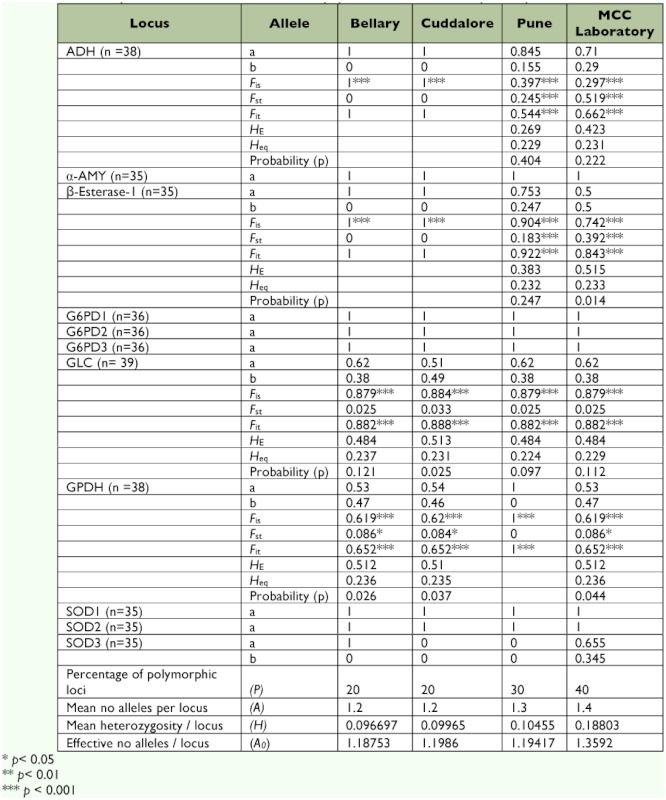
Allelic frequencies and *F* statistics for the four populations of *Cx. tritaeniorhynchus* species from India.

In DFA, to find out whether the clusters were significantly different from each other, Pillai's trace statistics (Harris 2001) were performed. Within-class covariance matrices were assumed to be equal and prior probabilities were taken into account while performing the discriminant analysis. A total of 334 measurements of 11 morphological characters were used in the multivariate analysis. Ellipses of probabilities are showed as circles.

The observed allelic frequencies for each population were used to estimate the mean number of alleles per locus (*A*), the effective number of alleles per locus (*A0*), percentage of polymorphic loci (*P*), and mean heterozygosity per locus (*H*) with respect to the Hardy-Weinberg expectation (Hartl and Clark 1997; Hedrick 2000). Agreement with Hardy-Weinberg proportions was tested using both *F*-statistics (Wright 1965, 1978; Weir and Cockerham 1984) and an χ^2^ test for goodness of fit with Levene's (1949) correction for small samples. To determine whether *F*_is_ and *F*it estimations for each locus were significantly different from zero, chisquare statistics [χ^2^ = *F* (2*n*) (*k -* 1)] were obtained, with *k*(*k* - 1)/2 degrees of freedom, where *n* was the sample size and *k* was the number of alleles (Weir 1990). To determine the significance of the *F*_st_ statistic per locus, the chi-square statistic [χ^2^ = (2*N*) *F*_st_(*k* - 1)] was used, with (*k -* 1) (*s -* 1) degrees of freedom, where *s* was the number of populations (Workman and Niswander 1970). As the χ^2^ test is likely to be unreliable when expected values are low (Sokal & Rohlf, 1995), the χ^2^ test was repeated with the genotypes pooled into three classes (i) homozygotes for the most common allele, (ii) heterozygotes for the most common allele, and (iii) other genotypes. Departures from Hardy-Weinberg were only considered significant if both χ^2^ tests were significant. The gene frequency data were analyzed using BOTTLENECK software (Piry et al. 1999) to assess evidence of recent bottlenecks. Deviations from expected heterozygosity were computed for each locus for each population. The infinite allele model (Kimura and Crow 1964) was used since it is the most appropriate for allozyme data (Piry et al. 1999). To determine the significance of deviations a two-tailed Wilcoxon sign-rank test (Luikart et al. 1998) was conducted. This method tested whether the expected gene diversity (*H*_E_) was higher than the expected equilibrium gene diversity (*H*_eq_), which was calculated from the observed number of alleles for each locus in each population under the assumption of mutation-drift equilibrium and the infinite allele model (Luikart and Cornuet 1998).

Morphological data on the four *Cx. tritaeniorhynchus* populations and from published literature on *Cx. pseudovishnui* and *Cx. vishnui* (Reuben 1968, Reuben et al. 1994) were used to construct the similarity and distance matrices with the Bay-Curtis index. The Bay-Curtis index is sensitive to out-lying values. Pairwise genetic similarity between the four populations were calculated according to the Nei's genetic similarity index (Nei 1978) and used in constructing the similarity and distance matrices. To compare the populations based on morphological and allozyme variation, the Neighbor Joining cluster analysis (Saitou and Nei 1987) was performed using Phylip software (Felsenstein 1989). Neighbor program (Phylip 3.68) software was used to construct the Neighbor joining trees. Bootstrap values were calculated using 1000 replications in the Seqboot program (Phylip software).

## Results

A quantitative examination of *Cx. tritaeniorhynchus* revealed that four populations were different with respect to morphological and allozyme variation.

### Phenotypic variation among the populations

The mean values of all the characters considered for morphological analysis showed significant difference among the four populations based on ANOVA (Table 2). The pairwise comparison of morphological characters using the unpaired *t*-test revealed that characters such as length of siphon, length of anal gills, and anal gill index were significantly different in all populations studied. While the siphon index of the Bellary and Pune populations was similar, the other populations showed significant differences from each other with respect to siphon index. The Pune and MCC laboratory populations had similar siphon/saddle ratios, while the other populations differed from each other. Pune populations were significantly different from the Bellary and Cuddalore populations with respect to siphon width. Saddle length for the Cuddalore and Pune populations was significantly different from the Bellary and MCC laboratory populations. MCC laboratory mosquitoes were significantly different in comb scale from all the other populations studied, and hence comb scale can be considered a unique character of the MCC laboratory population. All two tail p-values for the pairwise *t*-test were significant after Bonferroni adjustments, except for the denticles on the apical pecten tooth.

Between the populations of JE endemic areas (i.e. Bellary and Cuddalore) pecten teeth, width of anal gills, comb scale, and width of siphon were not significantly different, while remaining characters were significantly different even after Benferroni correction. Between populations of JE non-endemic areas, six characters — namely width of siphon, siphon saddle ratio, width of anal gills, pecten teeth, anal gill index and denticles on apical pecten tooth — were not significantly different, while the remaining characters were significantly different. When data for JE endemic and non-endemic region populations were pooled separately, the comparison showed that five characters — namely pecten teeth, denticles on apical pecten tooth, length of saddle, width of anal gills, and comb scales — did not differ significantly.

### Multivariate analysis of the populations

**Figure 2.**
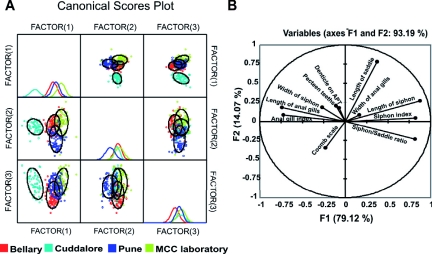
Discriminant factor analysis of morphological data. (A) Clusters of different populations. (B) Variables that discriminate between the clusters. High quality figures are available online.

PCA of the morphological analysis extracted five factors with eigen-values of more than one. Cumulatively, these factors explained 78.09% of the total variability in the data. The first factor (F_1_) explained 33.10% of the total variability, while the second factor (F_2_) explained 13.25% of the total variability; together the first two factors explained 46.35% of the total variability. Characters such as length of siphon, siphon index, siphon/saddle ratio, length of anal gills, and anal gill index showed high magnitude on F_1_. Length of siphon, width of siphon, length of saddle, length of anal gills, and denticle on apical pecten teeth showed high magnitude on F_2_. F_1_ was significantly different in all four populations of *Cx. tritaeniorhynchus,* whereas F_2_ was significantly different in Bellary and Pune populations. DFA could distinguish four significant clusters for Cuddalore, Bellary, Pashan and the MCC laboratory population (Pillai's trace = 1.583, *F* = 1.449, p < 0.0001) (Figure 2A). Characters that showed high factor loading on F_1_ included length of siphon, siphon index, and siphon/saddle ratio, while characters that showed high factor loading on F_2_ included length of saddle, length of siphon, and pecten teeth (Figure 2B).

### Allozyme variations among the populations

Allozyme electrophoresis resulted in clear and consistent staining for 7 enzymes encoded by 13 putative loci: ADH, α-Amy, G6PDH1, G6PDH2, G6PDH3, GPD, GLC, β-EST-1, β-EST-2, β-EST-3, SOD1, SOD2, and SOD3. All enzymes migrated anodally. A total of 18 alleles were detected from the four populations of *Cx. tritaeniorhynchus* (Table 4). The allele frequency analysis carried out on the four populations of *Cx. tritaeniorhynchus* revealed that Bellary, Cuddalore, Pune, and the MCC laboratory populations were monomorphic at loci α-Amy, SOD-1, SOD-2, G6PD-1, G6PD-2, and G6PD-3. Alleles β-EST-1^a^ and β-EST-1^b^ were detected in the Pune and MCC laboratory populations. β-EST-2 and β-EST-3 were only detected in the MCC laboratory population. All the β-EST bands (β-EST-1, β-EST-2, and β-EST-3) in the MCC laboratory population were weak, showing low esterase activity. Bellary, Cuddalore, and Pune populations showed prominent β-EST-1 activity. Bellary and Cuddalore populations shared monomorphic ADH and were considered to be encoded by a single monomorphic loci whereas, Pune and the MCC laboratory populations showed polymorphism in ADH with presence of ADH^a^ and ADH^b^ alleles. Two alleles, GLC^a^ and GLC^b^, were detected in all populations of *Cx. tritaeniorhynchus.* GPD^a^ and GPD^b^ alleles were detected in Bellary, Cuddalore, and the MCC laboratory, whereas the Pune population was monomorphic. SOD-3 loci were absent in the Cuddalore and Pune populations. SOD-3 was monomorphic in the Bellary population and SOD-3^a^ and SOD-3^b^ were present only in the MCC laboratory population. Isoenzyme profiles differed significantly between endemic and non-endemic populations. While enzymes like ADH and esterase were monomorphic in the case of endemic populations, they were polymorphic in non-endemic populations.

### Genetic variation within the population

Genetic variations were calculated in the four populations of *Cx. tritaeniorhynchus.* Mean number of alleles per locus were 1.2, 1.2, 1.3, and 1.4 for the Bellary, Cuddalore, Pune and MCC laboratory populations, respectively. Percentages of polymorphic loci were 20%, 20%, 30%, and 40%, respectively, and the mean heterozygosity per locus was 0.0879, 0.0905, 0.0950, and 0.1794, respectively. Effective numbers of alleles per locus were 1.187, 1.1986, 1.1941, and 1.359, respectively. *F* statistics for 10 loci (Table 4) described a high degree of geographic uniformity and suggested random mating between individuals within the populations. *F*_st_, *F*it, and *F*_is_ values were significantly different from zero. F_st_ values ranged between 0 and 0.519, suggesting significant genetic differentiation within these populations. F_is_ values ranged from 0–1 with most of the values closer to 1 (Table 4).

### Bottleneck effect

Table 4 shows the significant test results for a recent bottleneck by polymorphic loci in each population. For the MCC lab population, four loci showed significant differences in expected heterozygosity (*H*_E_) which was found to be higher than the expected heterozygosity at mutation equilibrium drift (*H*_eq_) (Table 4). For the Pune population, this was found for three loci, and for the Bellary and Cuddalore populations, this was found for two loci.

**Figure 3.**
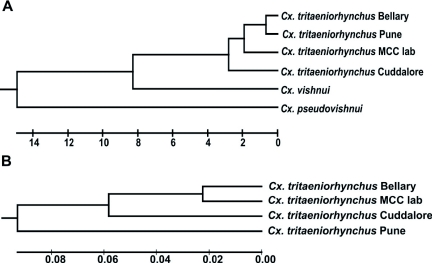
Neighbor joining tree showing: (A) the morphological relationship between four populations of *Culex tritaeniorhynchus, Cx. pseudovishnui,* and *Cx. vishnui* (B) genetic relationships among four populations of *Cx. tritaeniorhynchus* based on Nei (1978) pair-wise genetic distance. Each node is showing Bootstrap values based on 1000 replications. Bootstrap values below 50% are not shown. High quality figures are available online.

### Cluster analysis of morphological and allozyme data

The dendrogram obtained by the Neighbor joining clustering method revealed morphological similarities among the four different populations of *Cx. tritaeniorhynchus* and two outliers, *Cx. pseudovishnui* and *Cx. vishnui* (Figure 3A). All four populations of *Cx. tritaeniorhynchus* formed a single cluster, where the Bellary and Cuddalore populations showed high similarity followed by the MCC laboratory and Pune populations. The genetic similarities of the four populations depicted high similarity between the Bellary and Cuddalore populations, followed by the MCC laboratory and Pune populations (Figure 3B). Clusters obtained from morphological data and allozyme variation showed that populations endemic to JE infections were highly similar.

## Discussion

The effects of geographical and environmental events on morphological and genetic structure of local populations of *Cx. tritaeniorhynchus* were analyzed in this study. Using multivariate analysis of morphological characters, a significant morphological differentiation was observed in four populations of *Cx. tritaeniorhynchus* (Figure 2A). Principal component analysis and discriminant factor analysis of the data suggested that siphon, saddle, and anal gills related variables were the most important distinguishing characters (Figure 2B). Length and shape of larval siphon were considered to differentiate between species of *Cx. vishnui* group. Siphon index, considered a key character in mosquito taxonomy, was highest (13–14) in the Cuddalore population as compared to the remaining *Cx. tritaeniorhynchus* (9–10) populations studied. Siphon index of the Cuddalore population (13–14) was also greater than *Culex bankensis* (4–5), *Cx. pseudovishnui* (6–10), and *Cx. vishnui* (6–8). The Bellary and Cuddalore populations had shorter anal gill lengths as compared to the Pune and MCC laboratory-reared populations. The causes of morphological differences between populations are often quite difficult to explain. Phenotype is under the double control of environmental conditions and genotype, but morphological changes can be rapid when different environmental conditions occur (Klepaker 1993). The transition of siphon length and anal gill length in Cuddalore from the rest of the populations is hypothesized to be a consequence of selection due to environmental conditions.

Heterozygocity was an important parameter in determining the genetic variability. At the population level, genetic diversity of *Cx.* *tritaeniorhynchus* in the MCC laboratory population was higher (*A* = 1.4, *P* = 40 and *A0 *= 1.35 *H* = 0.188; Table 4) than the three field populations. However, the levels of genetic diversity were relatively similar when compared within the three field populations (Table 4). The mean heterozygosity per allele of *Cx. tritaeniorhynchus* populations (Table 4) was similar to the average values found in other diptera (Graur 1985). The highest genetic variability was found in the MCC laboratory population (0.1794), whereas the Bellary population showed the lowest genetic variability (0.087). The genetic variability in the MCC laboratory populations may have been due to isolation from the environment as these colonies were maintained in the laboratory for the past 12 years. Overall genetic identity within *Cx. tritaeniorhynchus* field populations from Bellary, Cuddalore, and Pune were high, 0.87(±0.299)– 0.96(±0.091), and these identities were markedly higher than the value of 0.85, suggested by Avise (1975) as the lower limit for conspecific populations. The genetic identity between the Bellary and MCC laboratory populations was found to be much higher 0.96(±0.091) than all the populations studied. Comparisons of allozyme analyses of *Cx. tritaeniorhynchus* showed higher homogeneity between the four populations, with F_is_ values ranging from 0.61 to 1 (Table 4) and Nei genetic identities ranging from 0.73(±0.302) to 0.96(±0.091). In the case of *Cx. tritaeniorhynchus* populations, there were single groups in the dendrogram. In concordance with high levels of heterozygosity, the BOTTLENECK test results (Table 4) indicated an excess of heterozygosity relative to allele numbers at several of the gene loci studied (Piry et al. 1999), which indicates that founder effects (bottlenecks) may have played a role in the history of the species.

Insecticide resistance complicates the role of mosquitoes in pathogen transmission because it limits the option for vector control. Members of *Culex* family have a notorious reputation for developing resistance to insecticides, including organophosphates, carbamates, and pyrethroids (Bisset et al. 1991, Ben Cheikh et al. 1998, Chandre et al. 1998, Kasai et al. 1998). All the β-EST bands (β-EST-1, β-EST-2, and β-EST-3) in the MCC laboratory population were weak showing low esterase activity. The Bellary, Cuddalore, and Pune populations showed prominent β-EST-1 activity. The presence of high activity esterase enzymes in the Bellary, Cuddalore, and Pune populations suggest the evolution and spread of resistance genes among populations across India. The residual spraying of insecticides in Bellary and Cuddalore has been ineffective at suppressing *Cx. tritaeniorhynchus.* Pune is non-endemic to JE but has long history of malaria vector control programs. It is possible that *Cx. tritaeniorhynchus* has been exposed to chemicals used for vector control and, hence, has acquired the high esterase activity. These observations clearly indicate that in the future different control measures will be required to reduce the *Cx. tritaeniorhynchus* populations.

Allelic frequency is one of the elements important in characterizing a population's genetic state. The higher the number of alleles per locus, the higher the adaptation possibility of a population. The allelic frequency analyses carried out in three field populations of *Cx. tritaeniorhynchus* revealed that the Pune population has more adaptation possibilities than the other two field populations. Due to urbanization, environmental conditions such as pollution, water quality, and air quality in Pune are different than in Bellary and Cuddalore. These environmental conditions might be playing a crucial role in the adaptability of the Pune population.

Comparison of non-endemic versus endemic areas of JE infection will help in better understanding the structure of *Cx. tritaeniorhynchus* populations. Better knowledge of population structures may benefit existing JE control strategies. Adaptability, genetic differentiation, heterozygosity, genetic identity, and bottleneck effects provide insights into population structure. In endemic areas of JE infection, little genetic differentiation was observed, whereas in non-endemic areas moderate genetic differentiation was observed. Adaptability was higher in non-endemic areas compared to endemic areas of JE infection. Excess of heterozygosity relative to allele number was observed in more loci of non-endemic areas compared to endemic areas of JE infection. These results clearly indicate that populations of *Cx. tritaeniorhynchus* in non-endemic areas have higher adaptability compared to endemic areas of JE infection. In Neighbor joining cluster analysis, populations of endemic JE infection areas showed high morphological and genetic similarity. Additional studies on genetic makeup and population dynamics will help in designing better vector control and antiviral strategies.

The null hypothesis, that there is no significant difference between morphometry and isoenzyme profile, was rejected. JE endemic and JE non-endemic populations differed significantly in characters related to siphon and anal gills. Isoenzyme profiles differed significantly between JE endemic and JE non-endemic populations. While enzymes like ADH and esterase were monomorphic in the case of JE endemic populations, they were polymorphic in JE non-endemic populations.

In conclusion, the traditional multivariate morphometry employed during the present study demonstrated its usefulness in distinguishing *Cx. tritaeniorhynchus* populations. It is important to point out that this is the first study to use multivariate analysis of morphological characters and allozyme analysis to resolve the population differences of *Cx. tritaeniorhynchus* in India. Results from this study illustrate the complexity of population variation in the mosquito *Cx. tritaeniorhynchus.* The allelic frequencies, *F* statistics, and Nei's genetic identity values showed that genetic differences between populations were small, but significant. The detected morphological and phenotypic variation may be related to differential environmental conditions such as temperature, food availability, and water quality. It is also known that in small populations, selection can often operate to influence the characters. The same characters, however, show that the environment confers adaptive morphological changes on organisms giving false morphological variations, suggesting the presence of stable variants. Efforts in correlating vector competence and its selection vis-à-vis morphological and genetic variation will be of great importance. It would be interesting to test whether microsatellites, which are known to give a closer estimation of heterozygosity at large-scale levels than allozymes, would be more useful in resolving the population structure of *Cx. tritaeniorhynchus.*

## **Abbreviations**

ADH,: alcohol dehydrogenase;
α-Amy,: amylase;
β EST,: β-Esterase;
DFA,: discriminant factor analysis;
G6PDH,: Glucose-6-phosphate dehydrogenase;
GLC,: Glucose-1 dehydrogenase;
GPD,: Glycerol 3-Phosphate dehydrogenase;
MCC,: Microbial Containment Complex;
PCA,: principal component analysis;
SOD,: superoxide dismutase

